# Understanding sex differences in long-term outcomes after a first episode of psychosis

**DOI:** 10.1038/s41537-020-00120-5

**Published:** 2020-11-20

**Authors:** Rosa Ayesa-Arriola, Víctor Ortíz-García de la Foz, Esther Setién-Suero, María Luz Ramírez-Bonilla, Paula Suárez-Pinilla, Jacqueline Mayoral-van Son, Javier Vázquez-Bourgon, María Juncal-Ruiz, Marcos Gómez-Revuelta, Diana Tordesillas-Gutiérrez, Benedicto Crespo-Facorro

**Affiliations:** 1 Department of Psychiatry, Marqués de Valdecilla University Hospital, IDIVAL. School of Medicine, University of Cantabria, Santander, Spain; 2 CIBERSAM, Centro InvestigaciónBiomédicaen Red Salud Mental, Madrid, Spain; 3 Hospital Universitario Virgen del Rocío, Department of Psychiatry, Universidad de Sevilla, Sevilla, Spain; 4 Instituto de Investigacion Sanitaria de Sevilla, IBiS, Sevilla, Spain; 5 Sierrallana Hospital, Department of Psychiatry, IDIVAL, School of Medicine, University of Cantabria, Torrelavega, Spain

**Keywords:** Psychosis, Psychosis

## Abstract

While sex differences in schizophrenia have long been reported and discussed, long-term sex differences in outcomes among first episode of psychosis (FEP) patients in terms of the efficacy of Early Intervention Services (EIS) has been an under-explored area. A total of 209 FEP patients (95 females and 114 males) were reassessed after a time window ranging from 8 to 16 years after their first contact with an EIS program (PAFIP) that we will call the 10-year PAFIP cohort. Multiple clinical, cognitive, functioning, premorbid, and sociodemographic variables were explored at 1-year, 3-year and 10-year follow-ups. At first contact, females were older at illness onset, had higher premorbid adjustment and IQ, and were more frequently employed, living independently, and accompanied by a partner and/or children. Existence of a schizophrenia diagnosis, and cannabis and alcohol consumption were more probable among men. During the first 3 years, women showed a significantly better response to minimal antipsychotic dosages and higher rates of recovery than men (50% vs. 30.8%). Ten years later, more females continued living independently and had partners, while schizophrenia diagnoses and cannabis consumption continued to be more frequent among men. Females also presented a lower severity of negative symptoms; however, functionality and recovery differences did not show significant differences (46.7% vs. 34.4%). Between the 3- and 10-year follow-up sessions, an increase in dosage of antipsychotics was observed. These results suggest that the better outcomes seen among women during the first 3 years (while they were treated in an EIS) were in the presence of more favourable premorbid and baseline characteristics. After an average period of 10 years, with the only difference being in negative symptoms course, outcomes for women approximated those of men, drawing particular attention to the increase in dosage of antipsychotic medication once FEP patients were discharged from the EIS program towards community-based services. These findings help to pose the question of whether it is advisable to target sexes and lengthen EIS interventions.

## Introduction

It is commonly believed that women with schizophrenia have better disease courses and better overall outcomes than men. A substantial proportion of male patients have poor long-term outcomes, namely, low-level functioning, unemployment, and substance abuse^1^. A later age-at-onset for females has been suggested as a factor involved in sex differences in incidence and course of illness^2^. However, Seeman et al.^3^, reported that women’s better outcomes declined over time and approached those of men. Further investigation is thus merited, particularly given the marked heterogeneity previously reported^4^ in functional outcomes among first episode of psychosis (FEP) patients, the possible design bias towards samples with poor outcomes^5^, and the lack of long-term studies on sex perspectives.

The studies that explored short-term and long-term (8 years or more considering the Morgan et al.^5^ definition) sex-outcome differences in FEP patients have produced mixed results. A study carried out in Hong Kong with young people suffering from an FEP^6^ found notable sex differences in functional outcomes, specifically in terms of higher levels of functioning in women (i.e., higher proportion of fulltime employment) within the initial 3 years of Early Intervention Services (EIS). However, in a recent observational study, Dama et al.^7^, found that sex differences in outcomes among patients treated over 2 years in an EIS for psychosis in Canada could be largely affected by the disparity of other factors that exist between the two sexes (i.e., childhood and early adolescent premorbid adjustment, and age at onset of psychosis). In the Danish OPUS trial, designed for 2-, 5-, and 10-year follow-ups, it was found that almost 30% of all FEP patients improved their outcomes between 5-year and 10-year follow-ups, indicating that improvement is still possible late in illness course^8,9^. These findings were similar to those reported in AESOP-10 (Etiology and Ethnicity in Schizophrenia and Other Psychosis 10-year follow-up), wherein three course and outcome domains (clinical, social, and service use) were worse for men^5^. Morgan et al.^5^, noted that symptom remission and recovery are more common than social re/integration following an FEP. But both OPUS and AESOP cohort studies suggest that short-term and long-term outcomes may differ. To the best of our knowledge, no study has specifically explored long-term sex differences in FEP patients after being discharged from an EIS for psychosis.

The aims of the present study were to explore in detail long-term sex differences among FEP patients through several outcome measures (clinical, functioning, cognition, and treatment); and to confirm/disconfirm if the positive effects achieved through treatment in an EIS may similarly last long-term for males and females once they are transferred to regular care. We hypothesized that after an average time of 10 years after an FEP, outcomes in women and men would be very similar.

## Results

**Fig. 1:**
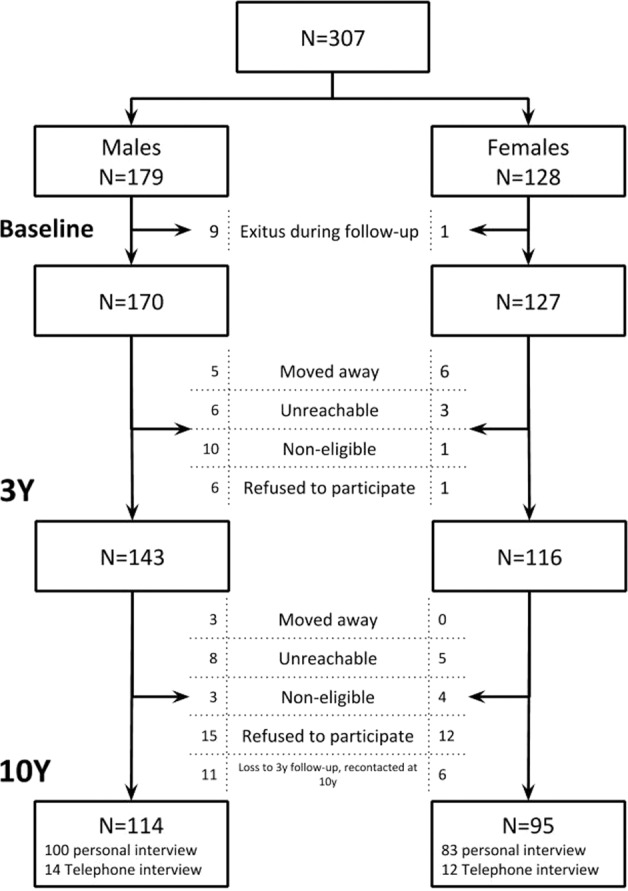
Flow diagram for study participants.

Between 2001 and 2008, 307 FEP patients were assessed for baseline PAFIP measures. Of these, 297 were re-tested at 1-year follow-up, and 259 at 3-year follow-up (See the flowchart of the study in Fig. 1). From the initial sample, 209 were tested after a time window ranging from 8 to 16 years (183 by in-person interview and 26 by telephone). Non-completers significantly had lower education level (61% vs. 44% elementary studies; *p* = 0.005), lived in urban areas (82% vs. 68%; p = 0.011), were unemployed (54% vs. 38%; *p* = 0.012), and were cannabis users (56% vs. 36%; *p* = 0.001). On the contrary, completers presented more severe depressive symptoms at baseline (F = 2.114; *p* = 0.035). A tendency towards higher participation in 10-year reassessment was found in women as well as more exitus among men (Information available in Supplementary Tables 1 and 2).

When comparing male and female FEP patients (95 females and 114 males) who completed the 10-year follow-up assessment, females were older at admission and at illness onset (31 years vs. 27 years; *p* < 0.001); had higher premorbid functioning during childhood, adolescence, and adulthood; presented higher premorbid IQ and education level; lived more frequently independently of parents (58% vs. 40%; *p* = 0.008), were employed (72% vs. 53%; *p* = 0.005), and had a partner (23% vs. 13%; *p* = 0.059) and children (40% vs. 14%; *p* < 0.001). Schizophrenia diagnosis (51% vs. 69%; *p* = 0.009), alcohol (33% vs. 66%; *p* < 0.001) and cannabis consumption (22% vs. 50%; *p* < 0.001) were more frequent among men. Ten years later, women continued living independently of parents (70% vs. 45%; *p* < 0.001) and more frequently had a partner (38% vs. 26%; *p* = 0.057). Schizophrenia diagnosis (63% vs. 84%; *p* = 0.002) tobacco (45% vs. 61%; *p* = 0.02) and cannabis consumption (4% vs. 12%; *p* = 0.038) continued to be more frequent among men (See Table 1).

**Table 1:** Comparison of baseline and 10-year follow-up characteristics between male and female FEP patients.

	Male		Female				
	*N* = 114		*N* = 95				
Characteristics	Mean	SD	Mean	SD	Statistic	Value	*p*
Age at admission (years)	27.4	7.5	31.7	9.6	*t*	−3.644	<0.001
Age at psychosis onset (years)	26.2	6.8	30.5	9.5	*t*	−3.662	<0.001
Duration of illness (months)	29.2	34.4	22.7	38.1	*t*	1.280	0.202
Duration of psychosis (months)	13.6	27.7	13.8	32.2	*t*	−0.056	0.956
Premorbid functioning	3.7	2.0	2.4	1.8	*t*	4.375	<0.001
Premorbid IQ	93.1	14.4	97.7	13.8	*t*	−2.040	0.043
Hospitalization (days)	21.9	18.1	18.8	14.2	*t*	1.086	0.280
*Baseline*	*N*	*%*	*N*	*%*	*Statistic*	*Value*	*p*
Diagnosis schizophrenia (yes)	79	69.3	49	51.6	*χ*²	6.854	0.009
Race (white)	112	98.2	94	98.9	Fisher		1.000
Urban area (yes)	79	69.3	65	68.4	*χ*²	0.019	0.891
Socioeconomic status of parents (low)	63	55.8	50	53.2	*χ*²	0.136	0.713
Education level (elementary)	65	57.0	27	28.4	*χ*²	17.196	<0.001
Living with parents (yes)	69	60.5	40	42.1	*χ*²	7.047	0.008
Single (yes)	96	84.2	62	65.3	*χ*²	10.084	0.001
Children (yes)	15	14.0	36	40.0	*χ*²	17.199	<0.001
Unemployed (yes)	54	47.4	27	28.4	*χ*²	7.838	0.005
Student (yes)	23	20.2	22	23.2	*χ*²	0.273	0.601
Tobacco (yes)	71	62.3	47	49.5	*χ*²	3.457	0.063
Cannabis (yes)	58	50.9	21	22.1	*χ*²	18.245	<0.001
Alcohol (yes)	76	66.7	32	33.7	*χ*²	22.573	<0.001
Hospitalization (yes)	77	67.5	56	58.9	*χ*²	1.655	0.198
*10Y*							
Diagnosis schizophrenia (yes)	84	84.0	53	63.9	*χ*²	9.781	0.002
Urban area (yes)	66	61.7	60	66.7	*χ*²	0.527	0.468
Living with parents (yes)	59	55.1	27	30.0	*χ*²	12.561	<0.001
Single (yes)	74	69.2	45	50.0	*χ*²	7.502	0.006
Children after FEP (yes)	8	7.5	12	13.3	*χ*²	1.838	0.175
Unemployed (yes)	35	32.7	22	24.4	*χ*²	1.624	0.202
Student (yes)	12	11.2	17	18.9	*χ*²	2.293	0.130
Tobacco (yes)	70	61.4	43	45.3	*χ*²	5.436	0.020
Cannabis (yes)	14	12.3	4	4.2	*χ*²	4.288	0.038
Alcohol (yes)	27	23.7	14	14.7	*χ*²	2.631	0.105
Hospitalization >1 (yes)	58	50.9	42	44.2	*χ*²	0.923	0.337

*IQ* intelligent quotient, *FEP* first episode of psychosis.

A group of 151 patients (85 female and 66 male) with clinical, functional, neuropsychological, and antipsychotic treatment information from assessments spanning the 10-year follow-up period were included in the results below.

### Positive, negative, and depressive symptoms in female and male FEP patients over 10 years: five consecutive follow-ups

Results revealed positive symptoms were similar at baseline and improved to about the same extent in males and females. Repeated measures analysis of covariance (ANCOVA) (with age at first contact as covariate) showed a significant within group by time effect (*F* = 21.883; *p* < 0.001). However, no significant between group by time effect was found.

Females showed more pronounced improvements (*F* = 8.135; *p* < 0.001) of negative symptoms than males. Significant between group by time (*F* = 9.33; *p* = 0.003) and within group by time differences were found at 6-week (*F* = 13.15; *p* < 0.001), 3-year (*F* = 6.131; *p* = 0.014) and 10-year (*F* = 5.983; *p* = 0.016) Scale for the Assessment of Negative Symptoms (SANS) assessments.

**Fig. 2:**
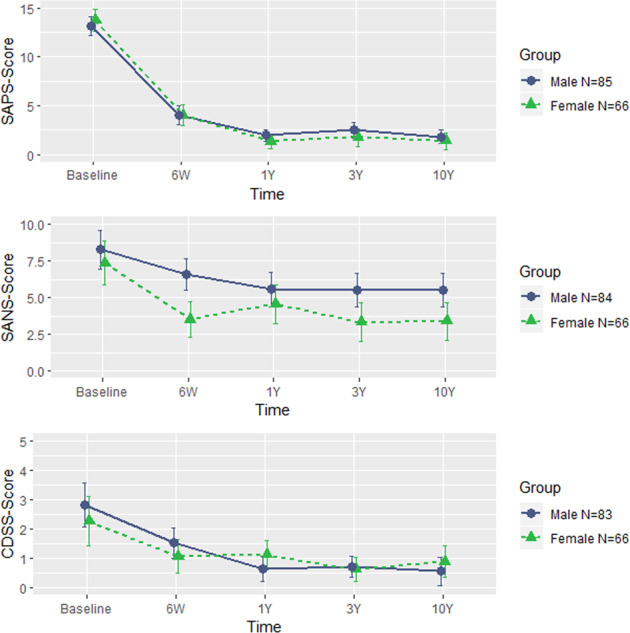
Clinical outcomes over follow-up in males and females.

Significant within group by time (*F* = 4.665; *p* = 0.032) but not significant between group by time effect was found for depressive symptoms, which evolved in parallel among male and female FEP patients (See Fig. 2).

### Functionality and global cognitive function in female and male FEP patients over 10 years

**Fig. 3:**
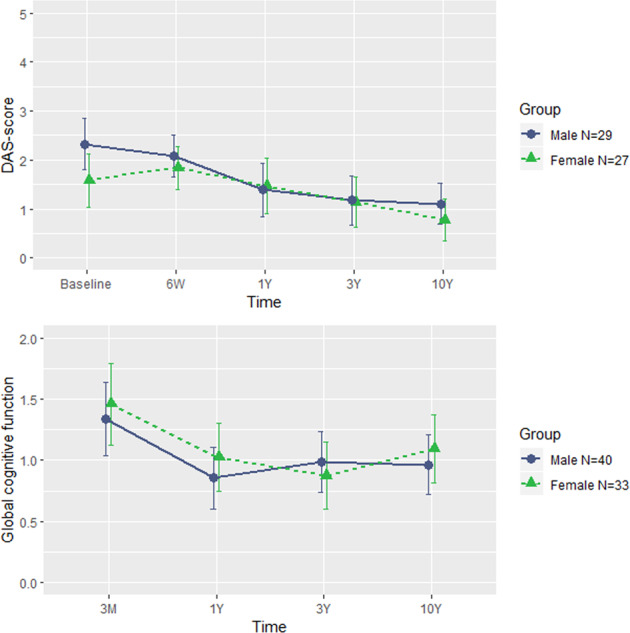
Functional and cognitive outcomes over follow-up in males and females.

Disability Assessment Scale (DAS) and global cognitive functioning (GCF) did not reveal either within group by time or between group by time effects. Exclusively years of education showed a significant group by time effect for GCF (*F* = 13.33; *p* = 0.001) (See Fig. 3).

### Sex differences in relapses and recovery across time

**Fig. 4:**
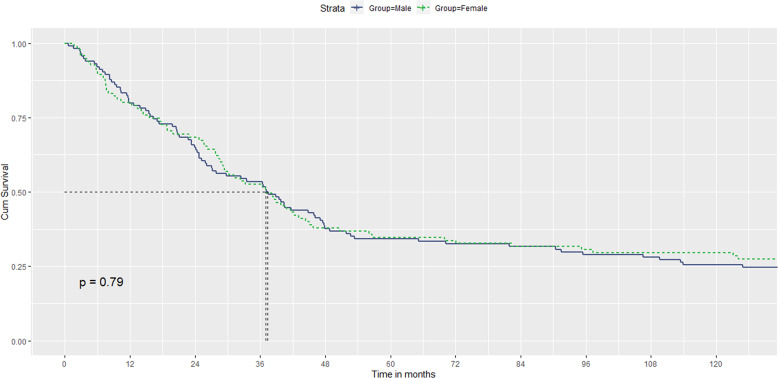
Survival curves of relapse over time in males and females.

Information on relapses was obtained for 142 patients (61 female and 81 male). Survival curves showed that most of the relapses occurred during the first 3 years of follow-up, with the curve flattening out owing to a lack of events between 3- and 10-year follow-ups. There was no significant difference (log-rank [Mantel–Cox]*χ*² = 0.07; *p* = 0.792) between female and male patients (See Fig. 4).

**Fig. 5:**
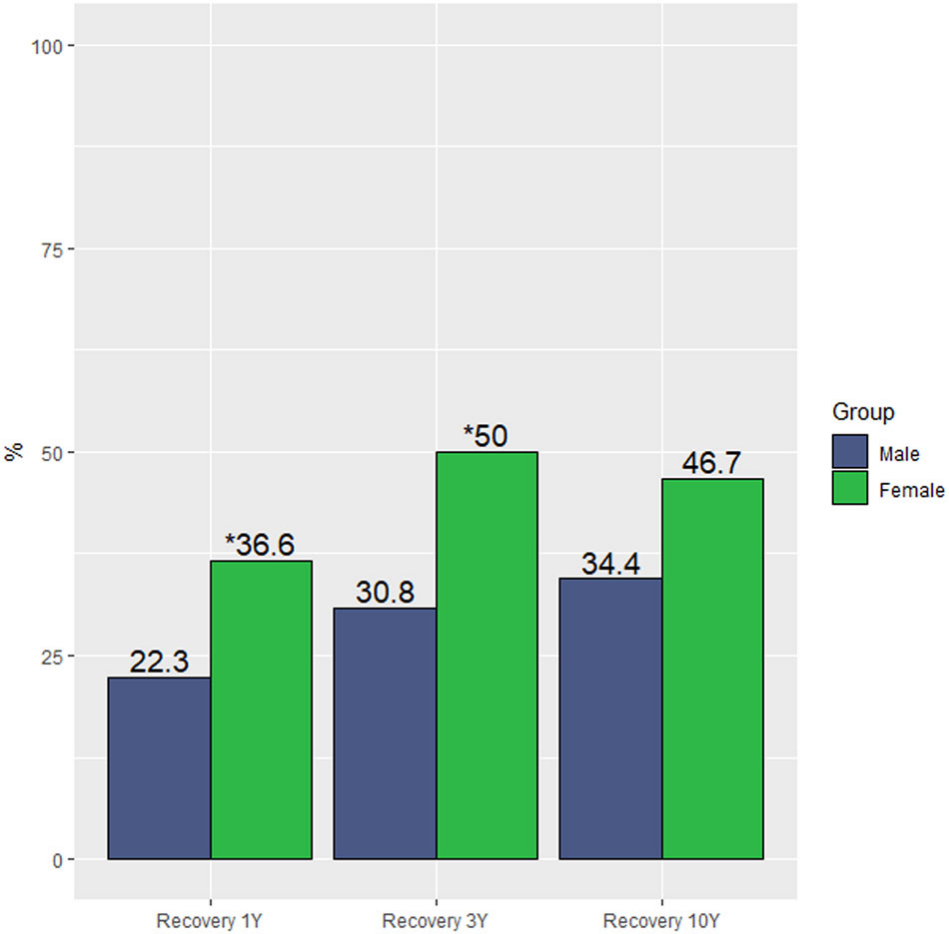
Recovery percentages in males and females over time.

Recovery information at 10-year reassessments was available for 171 patients (75 female and 96 male). Of these patients, 26 females (36.6%) and 21 males (22.3%) showed symptomatic and functional recovery at 1-year follow-up (*χ*² = 4.049; *p* = 0.044); 36 females (50%) and 28 males (30.8%) at 3-year follow-up (*χ*² = 6.234; *p* = 0.013); and 35 females (46.7%) and 33 males (34.4%) at 10-year follow-up (*χ*² = 2.656; *p* = 0.103). (See Fig. 5).

### Antipsychotic medication and extrapyramidal symptoms in female and male FEP patients: analyses of seven consecutive follow-ups

**Fig. 6:**
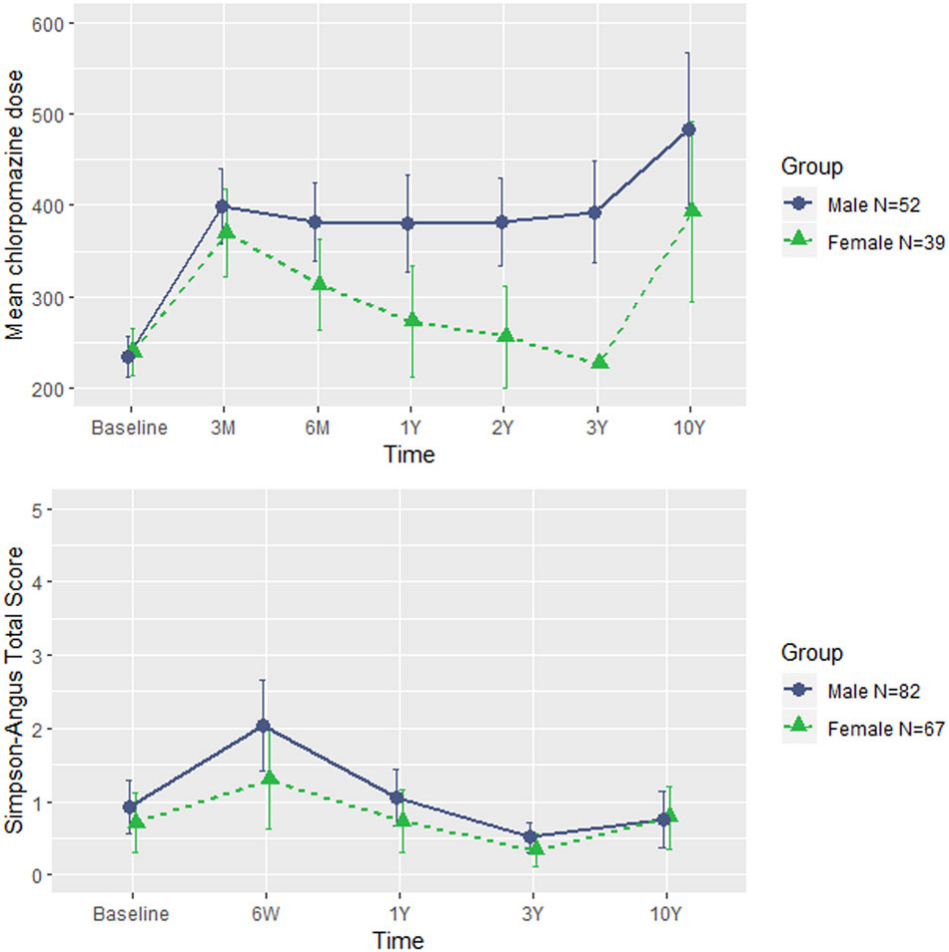
Chlorpromazine doses and Sympson–Angus scores over time in males and females.

Baseline, 3-month, 6-month, 1-year, 2-year, 3-year, and 10-year treatment information was available for 91 patients (52 male and 39 female). Repeated measures ANCOVA (age as covariate) revealed significant within group by time (*F* = 5.557; *p* = 0.021) and age by time (*F* = 6.46; *p* = 0.013), as well as between group by time effects (*F* = 12.184; *p* = 0.001). Significant differences in equivalent chlorpromazine dosage arose at 6-month (*F* = 4.27; *p* = 0.042), 1-year (*F* = 6.838; *p* = 0.01), 2-year (*F* = 11.533; *p* = 0.001) and 3-year (*F* = 14.841; *p* < 0.001) assessments, showing that females used lower therapeutic dosages. Minimal therapeutic dosages were reached at the 3-year follow-up, but between 3- and 10-year follow-up an increase in dosage was observed for both male and female patients. Males and females did not significantly differ on the occurrence of extrapyramidal symptoms (See Fig. 6).

## Discussion

This research examined sex differences in long-term outcomes after an FEP. Our results suggest that better outcomes for women found after 3 years of treatment at an EIS were underpinned by the presence of more favorable premorbid and baseline characteristics. After an average period of 10 years after treatment initiation, outcomes for women and men converged, with the exception of negative symptoms course. This draws particular attention to the increased dosage of antipsychotic medication for both sexes once they were discharged from the EIS program toward community-based services.

It could be said that women presented better functioning in terms of attaining milestones of adulthood such as completing school, having employment, leaving home, marrying, and having children. However, most of these circumstances were already present at illness onset, which might lead to the suggestion that women have an advantage in coping with difficulties associated with schizophrenia^3^. More women than men were partnered or married because women, whose illness begins later, often marry before their illness begins. Parenting was more common among women as well^8^. Those findings are consistent with the existing research of Hafner et al.^10^, supporting the mentioned advantages, such as higher rates of marriage and children, and lower rates of cannabis use. Seeman et al.^3^ found that despite women attaining better outcomes, there is a dependence on the social and cultural background of the study population. This is particularly the case in the exhibited maintenance of meaningful occupations, universally seen as positive and more frequent among females. In the same vein, both a higher age at illness onset of psychosis and better premorbid adjustment for women, which are favorable conditions for recovery, have been consistent findings^11^.

Having a first psychotic break at an older age is a positive prognostic factor because it allows higher premorbid achievements, in terms of greater knowledge and experience in the world before the illness, and time for the development of better social skills and greater resilience^12^. This is also confirmed in the present study concerning the higher education achievements and higher premorbid IQs in females, in line with a former study in our group^13^. Our study confirmed as well the replicated finding of equivalent cognitive impairment among male and female FEP patients^14^. It has been suggested that gender differences in neuropsychological impairments are evident in bipolar/mania and among depressive psychotic patients but with only minimal effects in schizophrenia^15^.

Women showed a marked recovery between the first and third year, but at 10 years the analysis of sex differences in functional recovery did not reach statistical significance. A similar finding was observed in a study carried out across diverse regions of the world^16^. Novick et al.^16^ found that in Central and Eastern Europe females showed higher rates of clinical remission at 36 months, with no sex differences in functional remission and recovery. Grossman et al.^12^, who studied recovery over a span of 20 years, found that women showed significantly better global functioning and higher percentages of recovery in the 2- and 10-year follow-ups. Compared with 41% of the men, 61% of the women with schizophrenia showed a period of recovery at some point during the 20-year period. Dama et al.^7^ suggested that some men may need more than 2 years of EIS to achieve good clinical outcomes and effectively improve their overall functioning. We suggest that patients receiving EIS beyond 3 years may continue to experience further improvements in negative symptoms and functioning, resulting in increased rates of recovery.

Women showed a consistent pattern of less severe and better course of negative symptoms than men. Overall, this result is in agreement with most of the published literature, wherein men with schizophrenia appear to have more negative symptoms and more severe clinical features, particularly in social withdrawal, substance abuse, and blunted or incongruent affects than female patients^17^. Hafner et al.^10^ proposed that the apparent vulnerability of men to schizoid characteristics might explain, at least partially, the increased negative symptom severity, earlier age of onset, and worse overall social functioning. Younger age at illness onset in men occurs concurrently with more negative symptoms^18^. Women have advantages over men in that their illness starts at later ages and their symptoms respond more quickly and more completely to available treatments^3^. However, even when age of onset was addressed by assessing a uniform sample (younger than 40 years old), and when there was no sex difference in premorbid adjustment, women showed a better course of illness^10^. In general, females have lower levels of negative symptom severity, lower rates of alcohol/substance abuse, and higher percentages of having a spouse/partner and independent living^16^. This may indicate that a healthy lifestyle, presence of a spouse/partner, and having children are protective roles for females against negative symptoms.

This study provides new evidence to what happens with antipsychotic treatment when patients are discharged from EIS programs towards routine outpatient mental healthcare services. Females particularly showed an increase in dosage. Effective doses of antipsychotics in women may have to be lower than recommended for men, especially with regards clozapine and olanzapine, and it is evident that some antipsychotic side effects, such as weight gain, are more worrying for women^19^. Women may be more vulnerable than men to adverse reactions because the recommended doses in the market are calculated for a 70 kg (154 lb) man^20^. The ability of women to respond to lower doses has been attributed to the effects of female hormones on the absorption and metabolism of antipsychotic medications, and to their relatively greater blood flow in the brain bringing more drug to receptors^21^. Brain structure, genetic and hormonal differences play a role on metabolic, cardiovascular, inflammatory, and immune parameters explaining sex differences in response to antipsychotics^20^. Seeman et al.^22^ suggested that the dosage intervals for women should be longer than for men. Furthermore, a recent review suggests that adjuvant estrogen may make treatment more effective and may reduce the dose of antipsychotics and, therefore, help to prevent side effects^23^. This applies to both sexes, but is especially applicable to women. Women require more thorough follow-up, including bone density scans, electrocardiograms, and mammograms. Meanwhile, the minimal effective dose of antipsychotics should always be selected. We found no differences in extrapyrimidal symptoms between male and female patients, which makes medication adjustments very important. Effective treatment means eliminating symptoms and improving function, but also the absence of side effects.

As expected, based on literature and in previous research in our group, females were older at illness onset and admission to the PAFIP program^4,14^. Mean age at onset for females was 4 years higher than that for males. This finding is consistent with the nationwide assessment of life time risks for mental disorders conducted in Denmark. Paying attention to sex- and age-specific incidence rates, and sex- and age-specific cumulative incidences of mental disorders treated in secondary care during a full life time, Pedersen et al.^24^ found that both sexes had almost identical incidence rates and cumulative incidences for schizophrenia during childhood and adolescence, but from 20 to 50 years of age, males had higher incidence rates and cumulative incidences. However, when Thorup et al.^25^, also in Denmark, extended the age range up to 71 years, they found that males had the highest incidence rates from age 17 to 40, whereas females had the highest incidence rates from age 50 to 68, confirming that male sex is a major risk factor for the development of schizophrenia but showing that risk for women increases with increasing age. They suggest that traditions in the diagnosis system (the narrowing of the diagnostic criteria leads to more women being excluded from a diagnosis of schizophrenia), and reluctance in some clinicians to use the diagnosis of FEP in elderly people lead to biases that should be avoided in the future.

To our knowledge, there are no other published longitudinal studies that have explored sex differences in such long-term outcomes among FEP patients after being in an EIS program. Most of the studies have been conducted in one of three ways, such that patients were sampled from chronic patients, were matched for sex, or were statistically controlled for sex in the analyses. To overcome this limitation, we focused our analyses on sex differences in FEP patients. Regretfully, the current analyses did not explore the relative contributions of biological (i.e., plasma level of estrogen), psychosocial (i.e., resilience) and environmental factors to the sex differences and their interplay, which clearly warrants further research. The choice of better measures to assess function, particularly concerning DAS, is not without its critics. DAS is a quite an outdated measure, consisting of a criteria that does not necessarily reflect the real functioning observed in outpatients. Meanwhile, the follow-up in the PAFIP cohort was scheduled for 3 years, at which point, the patients were discharged from our EIS to community mental health services. For the purpose of the present study, they were contacted within a time window ranging from 8 to 16 years after enrolling in the program. We cannot rule out the influence of information that was not recorded but could have affected outcomes. Finally, our findings concerning exitus corresponds with the results from a recent published nationwide cohort study that included patients admitted for the first time to hospital during 2000–2014 for schizophrenia or schizoaffective disorder in Finland^26^. Sommer et al.^26^ found that during the 10-year follow-up, mortality was lower in women, with fewer suicide and cardiovascular deaths, but more cancer deaths. A detailed exploration on exitus was not an aim of the present study, although it is an important issue to research in the future.

Neither sex has the monopoly on good outcomes. However, women have certain advantages over men in that their illness starts at a later age, providing them with more time to achieve goals in life, which in turn results in better outcomes over the first 3 years of treatment in specialized programs. Yet, these benefits for women appear to dissipate, particularly once they are discharged from an EIS towards community-based services, a transition that is accompanied by an increase in the doses of antipsychotic medication. This pattern helps to pose the question of whether it is wise to target the sexes and lengthen EIS interventions. Exploring long-term sex differences in outcomes among FEP patients could provide useful information to better understand and to improve sex specific approaches to treat psychosis.

## Methods

### Study design and setting

This paper is based on data obtained from PAFIP and PAFIP-10, which are incidence and 10-year follow-up studies, respectively, of all individuals with an FEP presenting for the first time to mental health specialists in the catchment area of Cantabria after February 2001 when a local EIS service for psychosis was established.

PAFIP was designed as an epidemiological prospective cohort study and a 3-year EIS program for FEP patients, conducted at the outpatient clinic and the inpatient unit at the University Hospital Marques de Valdecilla, Spain^27^. A multidisciplinary clinical team (three psychiatrists, two psychologists, one psychiatric nurse, and one social worker) provided 3 years of evidence-based phase-specific EIS for psychosis: low dose of antipsychotic medication while monitoring side effects, psychoeducation for patients and their families, and support for functional recovery based on return to normal daily activities (work/study) while expecting full recovery. Besides their routine visits in order to check symptoms and compliance with treatment, patients were contacted by telephone to schedule face-to-face appointments at 1- and 3-year follow-ups.

The PAFIP-10 study included FEP patients that were referred to the PAFIP program between February 2001 and December 2008. The patients constituting the 10-year follow-up PAFIP cohort were reassessed between June 2014 and August 2018. The patients included in the referred period were invited for a reassessment after a time window ranging from 8 to 16 years after initial presentation^4^.

### Ethical considerations

The PAFIP program was publicly funded by the regional Mental Health Services. Its research protocol was approved by the local Research Ethics Committee (CEIm of Cantabria) in accordance with international standards for research ethics (NCT0235832 and NCT02534363). Patients that met criteria for inclusion along with their families provided written informed consent for entry into PAFIP and for PAFIP-10 reassessment^4^.

### Subjects

The PAFIP-10 sample comprised all referrals to PAFIP between 2001 and 2008 that had been screened against the following inclusion criteria: age 15–60 years; living in the catchment area; experiencing their first episode of psychosis; no prior treatment with antipsychotic medication or, if previously treated, a total life time of adequate antipsychotic treatment of <6 weeks; meeting DSM-IV criteria (APA, 2000) for brief psychotic disorder, schizophreniform disorder, schizophrenia, or schizoaffective disorder. DSM-IV criteria for drug or alcohol dependence, intellectual disability, and having a history of neurological disease or head injury were exclusion criteria.

### Measures

This information has been previously reported in Ayesa-Arriola et al.,^4^. Baseline sociodemographic and clinical variables were obtained for a sample of 307 FEP patients (128 female and 179 male). Premorbid and sociodemographic information was recorded from interviews with patients, their relatives and from medical records on admission. Other baseline and demographic variables included: Sex, age, age at psychosis onset (defined as the age when the emergence of the first continuous psychotic symptom occurred), duration of untreated disease (DUI: defined as the time from the first nonspecific symptoms related to psychosis to the start of adequate treatment with antipsychotic medications, without return to the previous level functioning), duration of untreated psychosis (DUP: defined as the time from the first continuous psychotic symptoms to the start of appropriate treatment with antipsychotic medications), and premorbid social adjustment (measured using the Premorbid Adjustment Scale^28^, wherein the rating of 0, indicates “better”, and the rating of 6 denotes “worse”).

Baseline and 10-year characteristics were recorded, including: education level according to the Spanish educational system (“low” vs. “medium and high”), total years of education, socioeconomic status derived from the parents’ occupation (“low qualification worker” vs. “other”), living area (“urban” vs. “rural”, defined as more or <10,000 inhabitants, respectively), relationship status (“married/cohabiting” vs. “single/divorced/separated or widowed”), living status (“alone” vs. “other”), employment status (“employed” vs. “unemployed”), first-degree family history of psychosis based on subject and family reports (“yes” vs. “no”), as well as tobacco, alcohol and cannabis consumption (“yes” vs. “no”).

### Clinical and functional instruments

This study utilized clinical and functional data collected at four different points throughout the follow-up period. The same senior consultant psychiatrist (BC-F) interviewed patients at baseline, 1-, 3-, and at 10-year follow-up. Baseline diagnoses were assigned using the Structured Clinical Interview for DSM-IV (SCID-I)^29^ 6 months after the baseline visit. Based on follow-up information, diagnosis stability vs. change were considered at 10-year reassessment.

Clinical symptoms of psychosis were assessed by the SANS^30^ and the Scale for the Assessment of Positive Symptoms (SAPS)^31^. SAPS and SANS sub-scales were divided into positive (scores for hallucinations and delusions), disorganized (scores for formal thought disorder, bizarre behavior and inappropriate affect) and negative dimensions^32^ (scores for alogia, affective fattening, apathy and anhedonia). General psychopathology was assessed with the Brief Psychiatric Rating Scale (BPRS)^33^. Depressive symptoms severity was measured using the Calgary Depression Scale for Schizophrenia (CDSS)^34^.

Global functioning was measured by the DAS Spanish version^35^ on every assessment. The DAS was specifically designed for psychiatric populations and assesses the functioning status during the previous 30 days. This tool is a semi-structured interview that assesses several areas of functioning: self-care, social withdrawal, participation in the household, relationship with spouse or partner, occupational role and general interest. These areas are rated on a 6-point scale: 0, no disability; 1, minimum disability; 2, obvious disability; 3–5, serious to maximum disability.

A measure of GCF was calculated in accordance to previous methodology^36^. Scores on the seven cognitive domains fundamentally impaired in psychosis were converted to T-scores derived from a healthy comparison sample, and converted to deficit scores that reflected presence and severity of cognitive deficit on each cognitive domain. Deficit scores were then averaged to create the GCF measure.

Recovery, comprising symptomatic remission, and adequate psychosocial function at 1-, 3-, and 10-year follow-up, was determined by scores of 2 or less in the corresponding items of the SANS and the SAPS scales, as established by the remission work group^37^, and a score of 1 or less in the DAS scale^38,39^. Information about number of relapses during the 10 years were also considered, as well as mean antipsychotic daily doses (converted to chlorpromazine equivalents^40^) at seven different assessment points (baseline, 3 months, 6 months, 1 year, 3 years, and 10 years). Extrapyramidal symptoms were reported using Sympson–Angus Scale^41^ at baseline, 6 weeks, 1 year, 3 years, and 10 years.

### Statistical analysis

The Statistical Package for Social Science, version 19.0 (SPSS Inc., Chicago, IL, USA), was used for statistical analyses. All tests were two-tailed and the significance level was set at 5%.

Group differences for continuous variables were evaluated with *t* tests. Chi-squares were used for categorical variables. General Linear Models with repeated measures ANCOVAs were conducted to test longitudinal outcomes among male and female FEP patients. Bonferroni corrections were used to control for multiple comparisons. Post hoc comparisons presented in tables and figures for each dependent variable include only those cases with valid data on all of the variables at all time points.

Kaplan–Meier survival analysis, along with the log-rank test, was used to examine the relationship between relapses and time.

### Reporting summary

Further information on experimental design is available in the Nature Research Reporting Summary linked to this paper.

## Supplementary information

Supplementary Tables 1 and 2

Reporting Summary Checklist

## Data Availability

The data that support the findings of this study are available on request from the corresponding author R.A.A. The data are not publicly available as they contain information that could compromise research participant privacy/consent.

## Code availability

Code for data cleaning and analysis is provided as part of the replication package. It is available at 10.1017/S0033291719003854.
