# Supplementary material for: Understanding sex differences in long-term outcomes after a first episode of psychosis
Source: NPJ Schizophr. 2020 Nov 20;6:33. doi: 10.1038/s41537-020-00120-5 (PMC7679369; doi:10.1038/s41537-020-00120-5)
Supplement: Supplementary file 1 — Supplementary Tables 1 and 2 [file 41537_2020_120_MOESM1_ESM.pdf]

Supplementary Table 1. Comparison between FEP patients completers and non-completers of 10-year follow-up

	Completers N=209		Non-completers N=98		Total N=307				
Characteristics	Mean	SD	Mean	SD	Mean	SD	Statistic	Value	p
Age at admission, y	29.4	8.8	28.8	9.7	29.2	9.1	t	0.533	0.594
Age at psychosis onset, y	28.1	8.4	27.6	8.9	27.9	8.5	t	0.523	0.601
Duration of illness, mo	27.6	40.4	31.8	53.1	28.9	44.8	t	-0.760	0.448
Duration of psychosis, mo	15.0	35.2	14.4	29.5	14.8	33.4	t	0.134	0.894
Education, years	10.8	3.4	9.3	3.0	10.3	3.4	t	3.652	<0.001
Clinical									
SAPS	13.3	4.6	13.2	3.6	13.2	4.3	t	0.266	0.790
SANS	7.8	6.4	6.4	5.8	7.4	6.2	t	1.750	0.081
BPRS	62.0	13.2	61.1	11.4	61.7	12.7	t	0.555	0.580
CGI	6.2	0.7	6.3	0.7	6.2	0.7	t	-0.685	0.494
Positive dimension	7.4	2.4	7.5	2.4	7.5	2.4	t	-0.327	0.744
Negative dimension	6.0	5.8	5.1	5.6	5.7	5.8	t	1.221	0.223
Disorganized dimension	5.8	3.6	5.6	3.0	5.8	3.4	t	0.534	0.593
YMRS	13.0	5.9	14.2	7.4	13.4	6.5	t	-1.416	0.159
CDSS	2.6	3.4	1.8	2.7	2.4	3.2	t	2.114	0.035
DAS	1.2	1.4	1.2	1.4	1.2	1.4	t	-0.156	0.876
GAF	53.4	28.7	53.8	26.8	53.5	28.0	t	-0.061	0.951
	N	%	N	%	N	%	Statistic	value	p
Baseline									
Diagnosis									
Schizophrenia	128	61.2	56	57.1	184	59.9	χ2	0.467	0.494
Other schizophrenia spectrum diagnoses							χ2	1.501	0.826

Brief psychotic disorder	15	7.2	8	8.2	23	7.5			
Unspecified psychotic disorder	14	6.7	10	10.2	24	7.8			
Schizophreniform disorder	49	23.4	22	22.4	71	23.1			
Schizoaffective disorder	3	1.4	2	2.0	5	1.6			
Sex(male)	114	54.5	65	66.3	179	58.3	χ2	3.809	0.051
Race(white)	206	98.6	95	96.9	301	98.0	Fisher	0.920	0.388
Education level (elementary)	92	44.0	60	61.2	152	49.5	χ2	7.901	0.005
Socioeconomic status of parents (Not/Low qualified worker)	113	54.6	58	60.4	171	56.4	χ2	0.906	0.341
Urban area (yes)	144	68.9	81	82.7	225	73.3	χ2	6.447	0.011
Living with parents (yes)	109	52.2	60	61.2	169	55.0	χ2	2.219	0.136
Student (yes)	45	21.5	17	17.3	62	20.2	χ2	0.725	0.395
Single (yes)	158	75.6	79	80.6	237	77.2	χ2	0.953	0.329
Unemployed (yes)	81	38.8	53	54.1	134	43.6	χ2	6.371	0.012
Family psychiatric history (yes)	46	22.0	25	25.5	71	23.1	χ2	0.460	0.498
Hospital status inpatient (yes)	133	63.6	62	63.3	195	63.5	χ2	0.004	0.950
Tobacco use (yes)	118	56.5	61	62.2	179	58.3	χ2	0.919	0.338
Cannabis use (yes)	79	37.8	57	58.2	136	44.3	χ2	11.213	0.001
Alcohol use (yes)	108	51.7	59	60.8	167	54.6	χ2	2.237	0.135

BPRS: Brief Psychiatric Rating Scale, CDSS: Calgary Depression Rating Scale for Schizophrenia, CGI: Clinical Global Impression, SANS Scale for the Assessment of Negative Symptoms, SAPS Scale for the Assessment of Positive Symptoms, YMRS Young Mania Rating Scale, DAS Disability Assessment Scale, GAF Global Assessment of Functioning.

Supplementary Table 2. Comparison of reasons for attrition between male and female FEP patients

	Male N=179		Female N=128		Total N=307				
	N	%	N	%	N	%	Statistic	Value	p
Completed follow-up (Yes)	114	63.7	95	74.2	209	68.1	χ^2	3.809	0.051
Moved away (Yes)	8	4.5	6	4.7	14	4.6	χ^2	0.008	0.928
Unreachable (Yes)	14	7.8	8	6.3	22	7.2	χ^2	0.277	0.599
Non-eligible (Yes)	13	7.3	5	3.9	18	5.9	χ^2	1.523	0.217
Refused to participate (Yes)	21	11.7	13	10.2	34	11.1	χ^2	0.188	0.664
Exitus (Yes)	9	5.0	1	0.8	10	3.3	Fisher	4.271	0.050
