# Supplementary material for: Understanding sex differences in long-term outcomes after a first episode of psychosis
Source: NPJ Schizophr. 2020 Nov 20;6:33. doi: 10.1038/s41537-020-00120-5 (PMC7679369; doi:10.1038/s41537-020-00120-5)
Supplement: Supplementary file 2 — Reporting Summary Checklist [file 41537_2020_120_MOESM2_ESM.pdf]

nature research

Corresponding author(s): Rosa Ayesa-Arriola

Last updated by author(s): May 17, 2020

Reporting Summary

Nature Research wishes to improve the reproducibility of the work that we publish. This form provides structure for consistency and transparency in reporting. For further information on Nature Research policies, see our Editorial Policies and the Editorial Policy Checklist.

Statistics

For all statistical analyses, confirm that the following items are present in the figure legend, table legend, main text, or Methods section.

n/a	Confirmed	
☐	☒	The exact sample size (*n*) for each experimental group/condition, given as a discrete number and unit of measurement
☐	☒	A statement on whether measurements were taken from distinct samples or whether the same sample was measured repeatedly
☐	☒	The statistical test(s) used AND whether they are one- or two-sided<br>*Only common tests should be described solely by name; describe more complex techniques in the Methods section.*
☐	☒	A description of all covariates tested
☐	☒	A description of any assumptions or corrections, such as tests of normality and adjustment for multiple comparisons
☐	☒	A full description of the statistical parameters including central tendency (e.g. means) or other basic estimates (e.g. regression coefficient) AND variation (e.g. standard deviation) or associated estimates of uncertainty (e.g. confidence intervals)
☒	☐	For null hypothesis testing, the test statistic (e.g. *F*, *t*, *r*) with confidence intervals, effect sizes, degrees of freedom and *P* value noted<br>*Give P values as exact values whenever suitable.*
☒	☐	For Bayesian analysis, information on the choice of priors and Markov chain Monte Carlo settings
☒	☐	For hierarchical and complex designs, identification of the appropriate level for tests and full reporting of outcomes
☒	☐	Estimates of effect sizes (e.g. Cohen's *d*, Pearson's *r*), indicating how they were calculated

Our web collection on statistics for biologists contains articles on many of the points above.

Software and code

Policy information about availability of computer code

Data collection	SPSS 19, IBM
Data analysis	SPSS 19, IBM

For manuscripts utilizing custom algorithms or software that are central to the research but not yet described in published literature, software must be made available to editors and reviewers. We strongly encourage code deposition in a community repository (e.g. GitHub). See the Nature Research guidelines for submitting code & software for further information.

Data

Policy information about availability of data

All manuscripts must include a data availability statement. This statement should provide the following information, where applicable:

- Accession codes, unique identifiers, or web links for publicly available datasets
- A list of figures that have associated raw data
- A description of any restrictions on data availability

Provide your data availability statement here.

Field-specific reporting

Please select the one below that is the best fit for your research. If you are not sure, read the appropriate sections before making your selection.

☐ Life sciences ☒ Behavioural & social sciences ☐ Ecological, evolutionary & environmental sciences

For a reference copy of the document with all sections, see nature.com/documents/nr-reporting-summary-flat.pdf

Behavioural & social sciences study design

All studies must disclose on these points even when the disclosure is negative.

Study description	Long-term outcome in male and female first episode psychosis patients.
Research sample	A total of 209 first episode psychosis patients were reassessed at 10-year follow-up.
Sampling strategy	As presented in the flowchart, out of the 307 elegible subjects (179 males and 128 females), 209 were reassessed at 10-year follow-up (114 males and 95 females).
Data collection	During clinical interviews, scales and questionaries were completed by the psychiatrist, the psychologist, the nurse and the social worker; neuropsychological assessments were conducted by neuropsychologist.
Timing	Subjects included in the program From February 2001 to December 2008, reassessed between January 2016 and December 2018.
Data exclusions	*If no data were excluded from the analyses, state so OR if data were excluded, provide the exact number of exclusions and the rationale behind them, indicating whether exclusion criteria were pre-established.*
Non-participation	Non participation, as explained in the flowchart was due to: moved away, unreachability, non-elegibility, refuse to participate and exitus.
Randomization	Participants were randomly assigned to antipsychotic treatments at first contact with the study. However, this conditions was not taken into account for the purposes of the present study. Antipsychotics were converted into chlorpromazine equivalents.

Reporting for specific materials, systems and methods

We require information from authors about some types of materials, experimental systems and methods used in many studies. Here, indicate whether each material, system or method listed is relevant to your study. If you are not sure if a list item applies to your research, read the appropriate section before selecting a response.

Materials & experimental systems

n/a	Involved in the study	
☒	☐	Antibodies
☒	☐	Eukaryotic cell lines
☒	☐	Palaeontology and archaeology
☒	☐	Animals and other organisms
☐	☒	Human research participants
☐	☒	Clinical data
☒	☐	Dual use research of concern

Methods

n/a	Involved in the study	
☒	☐	ChIP-seq
☒	☐	Flow cytometry
☒	☐	MRI-based neuroimaging

Human research participants

Policy information about studies involving human research participants

Population characteristics	Male and female first episode of psychosis patients.
Recruitment	Individuals presenting for the first time psychotic symptoms in the catchment area of Cantabria were referred to the Program of Attention to Initial Phases of Psychosis (PAFIP).
Ethics oversight	The local Reseach Ethics Committee (CEIm of Cantabria) in accordance with international standars for research ethics aproved PAFIP and PAFIP-10 protocol (NTC0235832 and NTC02534363 respectively).

Note that full information on the approval of the study protocol must also be provided in the manuscript.

Clinical data

Policy information about clinical studies

All manuscripts should comply with the ICMJE guidelines for publication of clinical research and a completed CONSORT checklist must be included with all submissions.

Clinical trial registration	NTC02534363
Study protocol	PAFIP-10 STUDY
Data collection	Ten year follow-up clinical, neurocognitive and functioning information.
Outcomes	Outmes for clinical, neurocognitive and functioning at 10-year follow-up in male and females first episode of psychosis patients.
